# Outcomes at One-Year Post Anastomosis from a National Cohort of Infants with Oesophageal Atresia

**DOI:** 10.1371/journal.pone.0106149

**Published:** 2014-08-25

**Authors:** Benjamin Allin, Marian Knight, Paul Johnson, David Burge, on behalf of BAPS-CASS

**Affiliations:** 1 National Perinatal Epidemiology Unit, University of Oxford, Oxford, United Kingdom; 2 Department of Paediatric Surgery, John Radcliffe Hospital, Oxford University Hospitals NHS Trust, Oxford, United Kingdom; 3 Department of Paediatric Surgery, University Hospital Southampton NHS Foundation Trust, Southampton, United Kingdom; University of Florida, United States of America

## Abstract

**Background and Aims:**

We aimed to provide a contemporaneous assessment of outcomes at one-year post oesophageal atresia/tracheoesophageal fistula (OA-TOF) repair, focussing particularly on post-operative complications. It is generally accepted that oesophageal stricture is the most common complication and causes significant morbidity. We also aimed to assess the efficacy of prophylactic anti-reflux medication (PARM) in reducing stricture formation.

**Method:**

A prospective, multi-centre cohort study of all infants live-born with oesophageal atresia in the United Kingdom and Ireland in 2008/9 was performed, recording clinical management and outcomes at one year. The effect of PARM on stricture formation in infants with the type-c anomaly was assessed using logistic regression analysis.

**Results:**

151 infants were live-born with oesophageal atresia in the defined reporting period, 126 of whom had the type-c anomaly. One-year follow-up information was returned for 105 infants (70%); the mortality rate was 8.6% (95% CI 4.7–14.3%). Post-operative complications included anastomotic leak (5.4%), recurrent fistula (3.3%) and oesophageal stricture (39%). Seventy-six (60%) of those with type-c anomaly were alive at one-year with returned follow-up, 57(75%) of whom had received PARM. Of these, 24 (42%) developed a stricture, compared to 4 (21%) of those who had not received PARM (adjusted odds ratio 2.60, 95% CI 0.71–9.46, p = 0.147).

**Conclusions:**

This study provides a benchmark for current outcomes and complication rates following OA-TOF repair, with oesophageal stricture causing significant morbidity. The use of PARM appeared ineffective in preventing strictures. This study creates enough doubt about the efficacy of PARM in preventing stricture formation to warrant further investigation of its use with a randomised controlled trial.

## Introduction

With improvements in neonatal intensive care and surgical technique, mortality from oesophageal atresia with or without trachea-oesophageal fistula (OA-TOF) is now relatively low [1]–[3], and the majority of the burden of the disease is now accounted for by post-operative morbidity. Current estimates of post-operative morbidity, however, are often based on small, retrospective, single institution studies that are open to the influence of change in personnel or practice at the reporting institution, and as such do not always provide the most accurate overall representation of post-operative outcomes [4]–[7]. With the current move in the United Kingdom towards surgeon specific reporting of outcomes and increase in patient choice, it is important that there is accurate national data against which performances can be benchmarked [8]–[10].

A large proportion of morbidity post OA-TOF repair is accounted for by oesophageal stricture formation [7], and it is suggested that the presence of gastro-oesophageal reflux disease (GORD) increases the risk of this occurring [11]–[14]. In our previous report on the contemporary management of OA-TOF we identified that 54% of surgeons prescribed anti-reflux medication prophylactically to prevent both GORD and stricture formation [2]. Currently however, little evidence exists to suggest that this practice reduces the stricture rate [13], [15], [16].

Our study aims were therefore two-fold. Firstly, to report the outcomes at one year of age of a national cohort of infants with OA-TOF, and secondly, to investigate potential causative factors underlying the formation of oesophageal strictures post OA-TOF repair, focussing particularly on the role of PARM.

## Study Design, Setting and Participants

We performed a prospective, multi-centre cohort study of all infants live-born with OA-TOF in the United Kingdom and Ireland between 1^st^ of April 2008 and 31^st^ of March 2009. Infants were eligible for inclusion if they were treated at one of the twenty-eight paediatric surgical centres in the United Kingdom and Ireland and were diagnosed with any of the five Gross classifications of OA-TOF [17].

Cases were identified via the British Association of Paediatric Surgeons Congenital Anomalies Surveillance System (BAPS-CASS) as described by Owen et al. [18]. Patient identification was with the use of monthly case reporting cards sent to a designated responsible clinician in each paediatric surgical centre. In response to notification of a case via the reporting card, a detailed clinical questionnaire was sent to the responsible clinician. Returned data were coded and double entered into a customised database. A further clinical questionnaire was sent to responsible clinicians one year after the date of the initial operation in order to collect information on outcomes outside of the initial operative period for each identified infant. All data collected were anonymous, and missing or duplicated data were handled as described in previously published protocols [18].

Primary outcomes of interest were rates of mortality, stricture formation, anastomotic leak and recurrent fistula formation. A sub-group analysis of factors leading to stricture formation prior to one-year post-anastomosis was performed on infants with the Gross Type C anomaly. This sub-group was chosen, as they are the largest single anomaly, accounting for 86% of all OA-TOF anomalies in the United Kingdom and Ireland [2], and provided a more homogenous population for investigation of factors affecting stricture formation. As there is no consensus definition of oesophageal stricture, for the purpose of this study, infants were determined to have an oesophageal stricture if this was diagnosed by the responsible consultant, regardless of whether this was based on symptoms, endoscopic findings, or need for dilatation. The primary exposure of interest was the use of PARM. Patients were classed as having received PARM if at any point they had received any anti-reflux medication that was described as having a prophylactic intent, regardless of the duration of treatment or whether gastro-oesophageal reflux was later diagnosed.

Management and outcomes from birth to initial discharge has previously been documented for this cohort of infants [19]. Here, we are extending the analysis of the same cohort of infants to one year of age, and making a specific analysis of the role that PARM plays in stricture formation.

## Statistical Methods

Results are presented as rates or odds ratios with 95% confidence intervals or medians and interquartile ranges as appropriate. Mortality rates were calculated using the entire population of cases identified at index admission as the denominator. Rates of stricture formation, anastomotic leak and recurrent fistula formation were calculated using the number of infants who were alive at one year of age with follow-up information available as the denominator.

Factors considered likely to have a potential confounding influence on stricture formation were categorised into dichotomous variables. These included prematurity, delayed anastomosis, type of first operation (fistula ligation only, or primary anastomosis), development of a recurrent fistula or anastomotic leak, low birth weight, antenatal diagnosis, diagnosis of gastro-oesophageal reflux, delayed initiation of oral feeding, trainee surgeon performing the anastomosis, type of suture material used (absorbable vs. non-absorbable), sex, delayed initial presentation, use of post-operative ventilation, the presence of associated anomalies, and the ethnicity of the patient. Continuous variables were converted to dichotomous variables, with the 75^th^ percentile being used as the cut-off point for separation of the two groups. E.g. “Delayed initiation of oral feeding” was defined as ≥75^th^ percentile of time to initiation of oral feeding for all infants with Type-C anomaly who were alive with returned follow-up information at one year.

Logistic regression analysis was used to control for confounding effects of each independent variable. Potential confounding factors were inserted into the model in a forward stepwise manner in order of statistical significance of their effect on stricture formation. Independent variables were dropped from the model if they did not significantly affect the fit of the model, as defined by a p-value of >0.1 on likelihood ratio testing. The model was created including data from all infants alive at one year with follow-up information available for all independent variables of interest. All statistical analysis was performed using Stata version 11 (StataCorp. 2009. Stata: Release 11. Statistical Software. College Station, TX: StataCorp LP).

### Ethics Approval

This study was approved by the London Research Ethics Committee (study reference 07/H0718/92). All data collected were anonymous and patient consent was not required.

## Results – All Infants

Between 1^st^ of April 2008 and 31^st^ of March 2009, 151 live-born infants with OA-TOF were identified. Eighty-three per cent of identified infants had the type C anomaly. One year follow-up information was returned for 105 infants (70%). Infants with follow-up to one year were more likely to be white British, odds ratio 2.39 (95% CI 1.04–5.44, p = 0.0217), and less likely to have been transferred to a paediatric surgical centre after birth, odds ratio 0.268 (95% CI 0.064–0.851, p = 0.0149) than those without follow-up to one year (tables 1 and 2).

**Table 1:** Key characteristics of infants with complete follow-up information and those who have been lost to follow-up - all infants.

All Infants		Complete information available at one year follow-up (n = 105)*	Lost to follow-up (only baseline information available) (n = 46)*	p-value
**Ethnicity**	White British	79% (82)	61% (28)	0.0217
	Other	21% (22)	39% (18)	
**Transferred after** **birth**	Yes	74% (76)	91% (42)	0.0149
	No	26% (27)	9% (4)	
**Gestational Age** **at Birth (Weeks)**	<37	37% (39)	28% (13)	0.2729
	≥37	63% (65)	72% (33)	
	Median (IQR)	37 (35–39)	37 (36–40)	
**Birth Weight** **(g)**	<2500	41% (43)	43% (20)	0.772
	≥2500	59% (62)	57% (26)	
	Median (IQR)	2600 (2028–3010)	2605 (2170–2985)	
**Sex**	Male	53% (55)	57% (26)	0.6802
	Female	47% (49)	43% (20)	
**Associated** **anomalies**	Yes	56% (59)	58% (26)	0.8573
	No	44% (46)	42% (19)	
**Type of OA/TOF**	Type C	82% (86)	87% (40)	0.4421
	Other	18% (19)	13% (6)	

*Percentages calculated from the number of infants with complete data for the relevant variable.

**Table 2:** Key characteristics of infants with complete follow-up information and those who have been lost to follow-up – type-c infants only.

Type-C Infants only		Completeinformationavailable at oneyear follow-up (n = 86)*	Lost to follow-up(only baselineinformationavailable) (n = 40)*	p-value
**Surgical Approach**	Thoracoscopy	1% (1)	5% (2)	0.1885
	Thoracotomy	99% (85)	95% (38)	
**Initial operation**	TOF ligation only	10% (8)	7% (3)	0.711
	Primary repair	90% (76)	93% (37)	
**GOR diagnosed on** **index admission**	Yes	25% (21)	13% (5)	0.1099
	No	75% (63)	87% (35)	
**Index anti-reflux** **medication**	Yes	65% (56)	63% (25)	0.7754
	No	35% (30)	37% (15)	
**Stricture diagnosed** **on index admission**	Yes	6% (5)	15% (6)	0.0801
	No	94% (81)	85% (33)	

*Percentages calculated from the number of infants with complete data for the relevant variable.

The mortality rate at one year was 8.6% (13 infants, 95% CI 4.7–14.3). Of the 13 infants who died prior to one year, nine deaths occurred during the index admission, with a further four occurring after discharge but prior to one year of life. Three of the nine who died on initial admission were palliated and never underwent anastomosis. For the remaining ten who died, the median time from anastomosis to death was 114.5 days (IQR 47–216). Three infants were recorded as having died from overwhelming sepsis, two from complex cardiac defects, two as a result of other congenital malformations, and one secondary to each of tracheobronchomalacia, hydrocephalus and multicystic encephaloleukomalacia. The deaths of three further infants were undergoing investigation at the time of reporting, and cause of death was unknown. There were no infants reported as dying as a result of operative complications.

**Figure 1:**
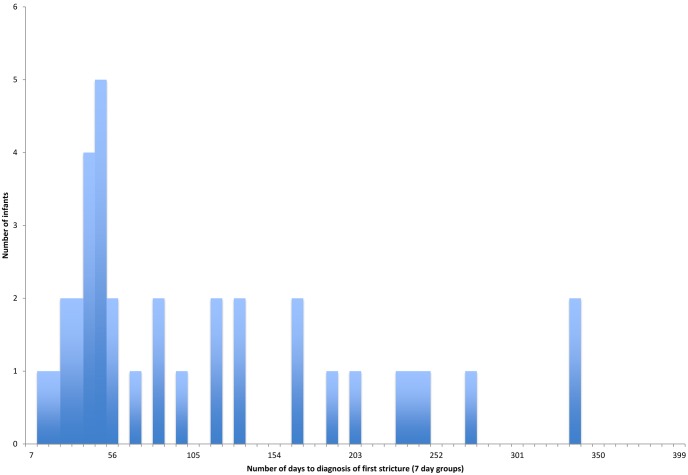
Time from anastomosis to diagnosis of first stricture.

The median time from anastomosis to initial discharge for infants surviving to one year was 15.5 days (IQR 11–39.5 days, n = 80 with complete information). For the 92 infants alive at one year with returned follow-up information, the recurrent fistula rate was 3.3% (3 infants, 95% CI 0.7–9.2), anastomotic leak rate was 5.4% (5 infants, 95% CI 1.8–12.2) and the stricture rate was 39% (36 infants, 95% CI 29–50). The median time to stricture formation for these 36 infants was 70.5 days (IQR 40.5–161.5 days) (Figure 1), and the median number of dilatations required in the first year was 3 (IQR 1–5 days, n = 30 with complete information).

### Infants with the Gross Type C anomaly

**Figure 2:**
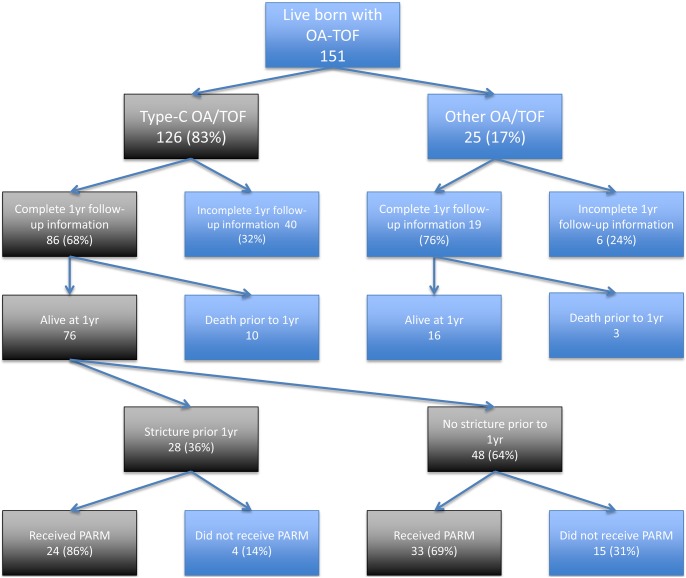
Flow diagram showing number of infants recruited and those who were lost to follow-up.

Follow up information was available at one year for 86 infants with the type C anomaly (68%), 76 of whom were alive. Of the 76 infants who were alive with returned follow-up data, 28 (36%) developed strictures prior to one year post-anastomosis. There were two further infants who had strictures reported, but these were diagnosed more than one year post-anastomosis, and therefore excluded from analysis. Of the 28 infants with reported strictures, 24 (86%) had received prophylactic anti-reflux medication, and four (14%) had not. Of the 48 infants who did not develop strictures, 33 (69%) had received prophylactic anti-reflux medication, and 15 (31%) had not. (OR 2.73, 95% CI 0.73–12.56, p = 0.0995) (table 3, and figure 2). The most commonly used anti-reflux medications were H2 receptor antagonists (73%), proton pump inhibitors (16%), motility agents (7%) and surface agents (4%).

**Table 3:** Effects of independent variables on the rate of stricture formation before and after controlling for confounding factors.

Characteristic		Stricture	No Stricture	Odds Ratio (95% CI)	P value	Adjusted odds ratio (95% CI)	Adjusted P value
**Prophylactic Anti-reflux Medication**	Yes	24	33	2.727 (0.73–12.56)	0.0995	2.60 (0.71–9.46)	0.147
	No	4	15				
**Prematurity (<37 weeks)** #	Yes	4	19	0.265 (0.059–0.97)	0.0256	0.32 (0.09–1.13)	0.079
	No	23	29				
**Delayed anastomosis**	≥61.5 hours	10	9	2.407 (0.73–7.94)	0.0995	2.69 (0.82–8.82)	0.101
	<61.5 hours	18	39				
**Type of first operation**	TOF ligation only	4	2	3.83 (0.50–44.39)	0.1146	*	*
	Primary anastamosis	24	46				
**Recurrent fistula or anastomotic leak**	Yes	4	2	3.83 (0.50–44.39)	0.1146	*	*
	No	24	46				
**Low birth weight**	<2500 g	8	21	0.514 (0.16–1.54)	0.1889	*	*
	≥2500 g	20	27				
**Antenatal diagnosis**	Yes	5	4	2.39 (0.46–13.13)	0.2151	*	*
	No	23	44				
**GOR diagnosed**	Yes	18	24	1.8 (0.63–5.30)	0.2270	*	*
	No	10	24				
**Delayed initiation of oral feeding** #	≥12 days	9	10	1.91 (0.56–6.32)	0.2338	*	*
	<12 days	17	36				
**Trainee primary operator**	Yes	3	10	0.456 (0.07–2.03)	0.2584	*	*
	No	25	38				
**Suture material** #	Absorbable	24	45	0.53 (0.07–4.33)	0.4564	*	*
	Non-absorbable	3	3				
**Sex**	Male	17	25	1.42 (0.50–4.11)	0.4654	*	*
	Female	11	23				
**Delayed presentation**	≥18 hours	9	12	1.42 (0.44–4.45)	0.5018	*	*
	<18 hours	19	36				
**Ventilated post-operatively**	Yes	27	45	1.8 (0.14–97.93)	0.6139	*	*
	No	1	3				
**Associated anomalies**	Yes	16	25	1.23 (0.43–3.50)	0.6695	*	*
	No	12	23				
**Ethnicity** #	White British	21	39	0.81 (0.22–3.17)	0.7182	*	*
	Other	6	9				

*Variable did not have a statistically significant effect on the fit of the model and was therefore excluded from the multivariate model.

# Information on Variable Missing for One Or More Infant.

Of the 76 infants with type-c anomaly who were alive at one year with follow-up information, 42 (55%) were diagnosed with GORD (either on clinical grounds, or as evidenced by pH monitoring or radiological investigation) by one-year post anastomosis. Of these 42 infants, 29 (69%) developed GORD despite receiving PARM. Of the 42 infants with a diagnosis of GORD at one year post anastomosis, 18 (42%) developed a stricture over the same time period, compared to 10 (29%) of the 34 who had not been diagnosed with GORD (OR 1.8, 95% CI 0.63–5.30, p = 0.2270) (table 3). Of the 42 infants with a diagnosis of GORD at one year post-anastomosis, eight (21%) of the 38 with information recorded had undergone fundoplication. 29 (51%) infants who received PARM developed GORD despite the use of prophylactic anti-reflux medication.

After evaluation of potential confounding factors, only prematurity and delayed anastomosis significantly affected the fit of the logistic regression analysis model. It was noted specifically that a later diagnosis of GORD (as determined by either clinical symptoms, radiological investigation or pH studies) had no impact on the outcome of interest (table 3). Following adjustment, the association between PARM and stricture formation remained non-statistically significant (aOR 2.60, 95% CI 0.71–9.46, p = 0.147). Prior to adjustment, prematurity appeared to be protective against stricture formation, with four (17%) of 23 infants born prior to 37 weeks developing strictures compared to 23 (44%) of 52 infants born after 37 weeks, odds ratio 0.265 (95% CI 0.059–0.97, p = 0.0256). However, there was no statistically significant association after adjustment (aOR 0.32, 95% CI 0.09–1.13, p = 0.079). Of the 19 infants with a delayed primary anastomosis, 10 (53%) developed strictures compared to 18 (32%) of the 57 without delayed anastomosis, aOR 2.69 (95% CI 0.82–8.82, p = 0.101).

## Discussion

We describe here one-year outcomes from a nationwide cohort study of infants with OA-TOF, against which individual centres can benchmark their practice, and on which counselling of parents can be based. Mortality post OA-TOF is relatively low at 8.6%, and median time to discharge is approximately two weeks post-anastomosis. The incidence of anastomotic leak is slightly lower than in other published series, potentially due to the fact that our analysis was based upon infants alive at one year with follow-up information, whilst the rate of recurrent fistula formation is broadly in keeping with previously published data [4], [20], [21]. The rate of fistula formation however may be slightly underestimated in our study as recurrent fistulae may continue to be detected for many years after our follow-up period of one year. Oesophageal strictures developed in nearly 40% of the operated population, with the majority being detected between 40 and 160 days post-operatively. Following diagnosis of a stricture, most infants required multiple dilatations in the first post-operative year.

After the introduction of proton pump inhibitors (PPIs) for the treatment of GORD in the general population, the incidence of oesophageal strictures appeared to decrease [13]. As a result, many paediatric surgeons began using anti-reflux medication prophylactically following OA-TOF repair. Since the initiation of this practice however, no robust evidence base has been generated to demonstrate benefit from it [15]. Our work in a population of infants with Type C OA-TOF that was heterogeneous in both its characteristics and the way that PARM was used suggests that the use of PARM does not appear to reduce the rate of stricture formation. It is likely however that there are multiple confounding factors influencing the relationship between PARM and stricture formation that we were unable to control for, particularly, unrecorded variation in intra-operative technique, and whether or not the anastomosis performed at initial surgery was under tension, both factors that many surgeons believe may increase the risk of stricture formation [21]–[23]. Another factor that may be related to stricture formation is the presence of a trans-anastomotic tube. However, this is not a relationship that has been robustly investigated previously, and due to the lack of infants treated without a trans-anastomotic tube in our cohort, it is not something we have been able to investigate. From an observational study such as ours, we have been able to highlight potential correlations, but not draw firm conclusions on causality.

Whilst we did not identify a statistically significant difference in stricture rate between those infants who received PARM and those who did not, there is a potential for this to have occurred because our study was underpowered. A lack of power is a difficulty encountered by many studies investigating conditions with a low incidence, and is frequently seen in paediatric surgical research. Data for our study was collected nationally, thus no additional cases could possibly have been added without extending the period of data collection, which we were unable to do, since this is a secondary analysis of previously collected data. The only practical way to increase the included population for studies such as this is therefore to develop international collaborations. We believe that by developing links with organisations such as the Canadian Paediatric Surgical Network (CAPSNet) [24], we can enhance our ability to provide a robust answer to many questions in paediatric surgery, including the efficacy of PARM in prevention of stricture formation.

Ranitidine is currently the most commonly used form of PARM following OA-TOF repair. It has however been shown to significantly increase the risks of infection, necrotizing enterocolitis and death in infants weighing less than 1500 g [25]. When taken in conjunction with this potential for harm, we believe that our study creates enough doubt about the efficacy of PARM in prevention of stricture formation to warrant further investigation of its use. A multinational cohort study as suggested above may provide a more precise estimate of benefit or harm. However, the logical next step would be a randomised controlled trial investigating the effect of PARM on stricture formation; the feasibility of this, given the small patient population, would need to be clearly established. The British Association of Paediatric Surgeons, with its national remit and participation from the majority of UK and Irish paediatric surgeons, would be the obvious body to take this forward.
